# Supplementary material for: Mechanical properties of raw bamboo fiber-reinforced phosphogypsum-based composite cementitious materials and their strengthening mechanism
Source: PLoS One. 2025 May 23;20(5):e0323011. doi: 10.1371/journal.pone.0323011 (PMC12101643; doi:10.1371/journal.pone.0323011)
Supplement: Attachment — Submitted filename: Response to Reviewers.docx [file pone.0323011.s002.docx]

Dear Solomon Oyebisi, PhD,

On behalf of my co-authors, we thank you very much for giving us another opportunity to revise our manuscript. We appreciate editors and reviewers very much for their positive and constructive comments and suggestions on our manuscript entitled "Mechanical properties of raw bamboo fiber-reinforced phosphogypsum-based composite cementitious materials and their strengthening mechanism" (PONE-D-24-56170).

We have revised the manuscript strictly in accordance with the suggestions of the editors and reviewers and have highlighted the revisions in red. We hope that this revised manuscript can meet the requirements for the paper's acceptance. The revised manuscript has been resubmitted to the journal of PLOS ONE according to the procedure.

Thank you for your thorough review of my paper submission. Regarding the raw data, the Origin file I submitted contains the raw data. If needed, I can send the raw data table to your email later.

We are looking forward to your positive response.

With kind regards.

Yours sincerely,

Corresponding author: Peng Liu

E-mail: liupeng00865032@163.com

Responses to reviewers' comments

Dear Editors and Reviewers,

Thank you for your letter and for the reviewers' comments concerning our revised manuscript entitled "Mechanical properties of raw bamboo fiber-reinforced phosphogypsum-based composite cementitious materials and their strengthening mechanism" (PONE-D-24-56170). Those comments are all valuable and very helpful for revising and improving our paper, as well as the important guiding significance to our researches. We have studied comments carefully and have made correction which we hope to meet with approval. Revised portion is marked in red in the paper. The main corrections in the paper and the response to their viewer's comments are as flowing:

Reviewer #1:

1. **Comment:** While the paper highlights the potential of bamboo fibers for reinforcing phosphogypsum, the novelty should be better articulated by comparing results with existing materials like polypropylene or steel fibers.

**Response:** Thank you for your careful review and valuable comments on the paper. Firstly, as noted in the introduction, I acknowledge that the list of adverse effects associated with various contemporary fibres, such as polypropylene and steel fibres, may not be exhaustive. To address this, a further comparison will be made between the performance of bamboo fibre and polypropylene fibre and steel fibre in terms of different performance indexes, as illustrated in a table:

Table 1

Performance of bamboo fibre with polypropylene fibre, steel fibre and polyvinyl alcohol fibre on different performance indicators

| Performance indicators | Bamboo fibres | Polypropylene fibres | Steel fibres | Polyvinyl alcohol fibres |
| --- | --- | --- | --- | --- |
| Mechanical performance | High tensile strength, good toughness, enhanced material crack resistance | Lower tensile strength for improved material resistance to cracking | Superior tensile strength to increase the overall strength of the material | Good tensile properties, but weak compared to steel fibres |
| Environmental benefit | Renewable Energy, biodegradable, low carbon footprint | Petroleum-based material, non-degradable, negative impact on the environment | High energy consumption, difficult recycling, higher carbon emissions | Environmentally friendly materials, biodegradable, low carbon footprint |
| Cost effectiveness | Low-cost, resourceful and particularly suited to large-scale production | Medium cost, widely used but costly in the long term | High cost, higher production and transport costs | Higher production costs, especially in large-scale utilisation |
| References | Zhang et al. (2020), Liu et al. (2021) | Silva et al. (2018), Mahmood et al. (2020) | Li et al. (2017), Mohamed et al. (2019) | Zhang et al. (2022), Chen et al. (2020) |

Secondly, within the ambit of the 'bamboo instead of plastic' research, the utilisation of bamboo fibre-reinforced phosphogypsum, as an emerging building material, has the potential to amalgamate the environmental properties of phosphogypsum with the elevated strength of bamboo fibres. In comparison to conventional polypropylene and steel fibre reinforcements, bamboo fibre-reinforced phosphogypsum exhibits distinctive advantages in terms of environmental sustainability, resource efficiency, and compatibility with biodegradable materials. In response to your suggestion, we will further clarify and mark in red the introductory paragraph, which will be reworked as follows:

To enhance the mechanical properties of phosphogypsum and broaden its construction applications, numerous studies have been conducted globally. However, practical engineering applications face challenges: steel fibers are prone to corrosion when exposed to air [18, 19], polypropylene (PP) fibers exhibit low tensile strength [20, 21], polyvinyl alcohol fibers are costly [22, 23], and glass and asbestos fibers pose health hazards [24, 25]. In contrast, plant fibers are natural polymer materials with specific chemical compositions [26, 27], drawing significant attention due to their safety, availability, biodegradability, and potential for sustainable regeneration [28-30]. Among these, bamboo fiber—a rapidly renewable plant fiber—exhibits mechanical properties comparable to traditional fiber-reinforced cement, significantly enhancing the toughness of composite cementitious materials [31, 32]. Moreover, bamboo fibre-reinforced phosphogypsum has been demonstrated to exhibit distinct advantages over conventional fibre-reinforced materials with regard to environmental friendliness, resource and sustainability, and compatibility with biodegradable materials.

2. **Comment:** The selection of the optimal bamboo fiber length (12 mm) and doping amount (1.0%) lacks a clear explanation of whether other combinations might provide comparable or better results.

**Response:** Thank you for your careful review. Regarding your question “The selection of the optimal bamboo fiber length (12 mm) and doping amount (1.0%) lacks a clear explanation of whether other combinations might provide comparable or better results.” we have revisited the experimental design and provided an explanation.

In the experiment, the variation in length of bamboo fibre was set at 4mm, 8mm, 12mm, and 16mm, with the fibre amount for each length set at four levels (0, 0.50, 1.0, 1.5wt%), as evidenced by the experimental results:

（1） A bamboo fibre length of 12 mm has been shown to optimise the properties of the composite while maintaining adequate mechanical strength and stability. The addition of longer bamboo fibres (16 mm) has been found to enhance the strength to a certain extent, however, it has also been observed to affect the dispersion of the fibres and the homogeneity of the material. In contrast, shorter fibres (4 mm) have been found to be ineffective in providing the desired reinforcing effect of bamboo fibres. Consequently, the 12 mm length is regarded as a balanced choice.
（2） At a doping level of 1.0 wt%, the bamboo fibres were found to enhance the mechanical properties of the composites to a significant extent, without exerting an excessive negative impact on the processing properties of the materials. Conversely, higher (1.5 wt%) or lower (0.50 wt%) doping levels resulted in a decline in mechanical properties.
（3） Following the experimental study, it was determined that the optimal mechanical properties of BFRGCs were attained with a bamboo fibre length of 12 mm and a doping level of 1.0 wt%, as illustrated below:

**Fig. 5.** Variation of flexural strength of BFRPGCs.

**Fig. 6.** Variation of compressive strength of BFRPGCs.

3. **Comment:** The one-way experimental design is appropriate but limits the exploration of interactions between parameters. A factorial design could provide deeper insights.

**Response:** Thank you for your careful review and valuable comments on our work. We appreciate your suggestions regarding the experimental design. We agree with your observation that “the one-way experimental design is appropriate but limits the exploration of interactions between parameters, and a factorial design could provide deeper insights.” Due to time and resource constraints in the current study, we chose a one-factor experimental design to focus on the effects of bamboo fiber length and dosage on the mechanical properties of phosphogypsum-based cementitious materials. This limited our ability to perform ANOVA or explore interactions between parameters. We understand that a factorial design would offer a more comprehensive understanding, particularly in terms of multi-parameter interactions. We apologize for not being able to implement a factorial design in this study. However, in future research, we plan to expand the experimental design to incorporate a factorial approach, which will allow us to better explore the interactions among factors and conduct the relevant statistical analyses. This will provide deeper insights into the combined effects of individual parameters on the experimental results.

4. **Comment:** The study does not mention the use of statistical tools to validate the significance of observed trends in mechanical properties. Adding ANOVA or regression analysis could strengthen the reliability of findings.

**Response:** Thank you for your careful review of our study. We greatly appreciate your questions regarding the statistical analysis tools (e.g., ANOVA and regression analysis). We fully recognize the value of these methods in verifying the significance of trends in mechanical properties and in enhancing the reliability of the study. In the current study, statistical methods such as analysis of variance (ANOVA) or regression analysis were not employed because our experimental design was based on a one-way approach, focusing primarily on the effect of each individual variable (such as the length of raw bamboo fibers or the amount of blending) on the mechanical properties. Although we performed multiple repetitions in our experiments and observed relatively consistent trends across the experimental groups, which led us to believe that the results were reliable, we did not use statistical tools to quantify the significance of these trends.

We apologize for this omission and fully acknowledge the importance of your suggestions in further enhancing the rigor and credibility of our study. For future research, we plan to adopt more comprehensive experimental designs, including multifactorial and factorial approaches, which will enable us to apply statistical tools (such as ANOVA and regression analysis) to validate the significance of the results and strengthen the overall credibility of our findings.

Once again, we are grateful for your valuable comments, which have provided crucial direction for the improvement of our future research. We will incorporate your suggestions into our experimental design and ensure that appropriate statistical analyses are conducted in future studies. We look forward to receiving your guidance again in the future.

5. **Comment:** The mechanical properties of control specimens are referenced but not thoroughly analyzed to demonstrate the incremental benefits of bamboo fibers.

**Response:** Thank you for your valuable suggestion. We appreciate your identification of the issues related to the analysis of the mechanical property control samples. We agree that this section requires further discussion to better highlight the positive effect of bamboo fibers. In the current study, while we did mention the mechanical properties of the control (blank) samples, the primary focus was on evaluating the impact of bamboo fibers on the mechanical properties of phosphogypsum-based cementitious materials. As a result, our description of the control samples was relatively brief. We now recognize that a more detailed analysis of the matrix strength without the addition of bamboo fibers is crucial to more clearly demonstrate the benefits provided by the bamboo fibers. We apologize for this omission and will provide a more comprehensive analysis of the control samples in the revised version.

Specifically, we will include a detailed data analysis of the control samples, focusing on mechanical property indices, and compare these with the samples containing bamboo fibers to better illustrate the gain effect of the bamboo fibers. This will not only make the results more thorough and persuasive, but will also clarify the actual contribution of bamboo fibers to the material properties.

Once again, we greatly appreciate your insightful comments. We have added the necessary analyses to the manuscript, and these additions are highlighted in red. The revised sections are provided below:

（1） Fig. 5 illustrates the variation in flexural strength of bamboo fiber-reinforced phosphogypsum composites (BFRPGCs) as a function of bamboo fiber length and doping amount. Without the addition of bamboo fibers, the 7-day and 28-day flexural strengths of PGCs were 4.31 MPa and 4.95 MPa, respectively. After the incorporation of bamboo fibers, the flexural strength of BFRPGCs gradually increased with the increase in fiber content, while the length of the bamboo fibers remained constant.
（2） Fig. 6 illustrates the relationship between the compressive strength of Bamboo Fiber Reinforced Geopolymer Composites (BFRPGCs), the length of bamboo fibers, and the amount of doping. Without the addition of bamboo fibers, the 7-day and 28-day compressive strengths of PGCs were 16.20 MPa and 23.43 MPa, respectively. After the addition of bamboo fibers, the 7-day compressive strength of BFRPGCs increased gradually with the increase in fiber content, provided that the length of the bamboo fibers remained unchanged and did not exceed 12 mm.
6. **Comment:** The SEM analysis is informative but would benefit from quantitative metrics, such as porosity measurements or fiber-matrix adhesion strength, to substantiate qualitative observations.

**Response:** Thank you for your valuable comment. We greatly appreciate your suggestion to incorporate quantitative metrics into the SEM analysis. While SEM images provide important qualitative information, we agree that including quantitative analyses (e.g., porosity measurements or quantification of fiber-matrix bond strength) would enhance the credibility of our findings and better support our observations. In this study, the SEM analysis focused on examining the microscopic morphology of the samples and describing the fiber-matrix interface. However, due to the limitations of the experimental design, we did not conduct quantitative measurements of porosity or fiber-matrix bond strength. We apologize for this limitation and recognize that your suggestions are essential for improving the rigor of our study.

As you pointed out, porosity is an important factor influencing the mechanical and adhesive properties of the phosphogypsum matrix. In future studies, we plan to extend our analysis by incorporating porosity measurements, tests of fiber-matrix bond strength, and other relevant quantitative metrics. These additional analyses will allow us to validate the trends observed in the SEM images more comprehensively and further strengthen the reliability of our conclusions.

Thank you again for your professional guidance. We have revisited the paper and made improvements to the SEM images. The revised version, with changes marked in red, is shown in the figure below:

**Fig. 9.** SEM images of BFRPGCs specimens at different doping levels.

7. **Comment:** The long-term performance and durability of BFRPGCs under environmental conditions (e.g., moisture, freeze-thaw cycles) are not addressed, which is critical for construction materials.

**Response:** Thank you for your valuable comments. We take your question regarding the long-term performance and durability of bamboo fiber-reinforced phosphogypsum-based cementitious materials (BFRPGCs) under environmental conditions (e.g., humidity, freeze-thaw cycles, etc.) very seriously. We fully agree that these factors are critical for the practical application of building materials.

In the current study, we only conducted short-term performance tests, primarily focusing on a 28-day curing period. The main objective of these experiments was to evaluate the initial impact of bamboo fibers on the mechanical properties of phosphogypsum-based cementitious materials and to provide a preliminary assessment of the material's performance. Due to the limitations of the experimental design, we did not address long-term durability aspects (e.g., freeze-thaw cycles, humidity effects, etc.) or environmental adaptability in depth. We apologize for this limitation and fully understand your concerns regarding the long-term performance and durability of the material. These factors are indeed crucial for the practical use of building materials, especially in varying environmental conditions. As such, we plan to extend our future research to include long-term durability testing, specifically focusing on the performance of BFRPGCs under the influence of humidity, freeze-thaw cycles, and other environmental factors. These follow-up experiments will allow us to more comprehensively assess the long-term stability and durability of bamboo fiber-reinforced phosphogypsum-based materials and provide stronger support for their practical application in engineering projects. In addition, we intend to adopt standardized durability testing methods (e.g., freeze-thaw cycles, hot and humid environment tests, etc.) in future studies to systematically evaluate the long-term performance of BFRPGCs. These data will complement the current study and offer essential insights into the sustainable use of bamboo fiber-reinforced phosphogypsum materials in the construction industry.

Once again, we appreciate your thoughtful comments. We will carefully consider your concerns about long-term performance in our revisions and plan to address this important issue in greater detail in subsequent studies.

8. **Comment:** The paper does not discuss potential challenges in scaling the laboratory findings to industrial applications, particularly with uniform fiber dispersion.

**Response:** Thank you for your valuable comments. We greatly appreciate your insightful suggestions regarding how to extend laboratory findings to industrial applications, particularly with respect to ensuring uniform fiber dispersion. We fully understand the concerns raised by the reviewers and sincerely apologize for not adequately addressing this topic in the original manuscript. We are grateful for your thoughtful feedback and will use this opportunity to further strengthen our paper.

The challenge of translating laboratory findings into practical industrial applications, especially with regard to achieving uniform fiber dispersion, is indeed a key aspect of this study. Raw bamboo fiber, being a natural fiber, is highly hygroscopic and hydrophilic, making the dispersion and homogeneity of the fibers crucial factors influencing the performance of phosphogypsum-based gelling materials during preparation. In the laboratory stage, we have improved fiber dispersibility to some extent by optimizing the fiber pretreatment process, including drying and manual dispersion. However, achieving effective and uniform dispersion of raw bamboo fibers into phosphogypsum-based gelling materials in industrial settings remains an area that requires further research and technological advancement.

We anticipate that the homogeneous distribution of fibers could be significantly improved in the future with the aid of industrial mixing equipment, such as high-shear mixers or ultrasonic dispersion systems. We will address these challenges and potential solutions in greater detail in the final section of the paper, and we will also highlight directions for future research. The revisions have been marked in red as follows:

Although this study demonstrated the positive effects of raw bamboo fibers on the properties of phosphogypsum-based cementitious materials, a critical challenge remains in translating these results from laboratory conditions to practical industrial applications, especially with regard to ensuring uniform fiber dispersion. Experimentally, we have improved fiber dispersion by optimizing the fiber pretreatment processes (e.g., drying, artificial dispersion, etc.). However, under industrial-scale production conditions, achieving uniform fiber distribution remains a significant challenge. Therefore, future research should focus on the use of efficient mixing technologies, such as high-shear mixers or ultrasonic dispersing equipment, to further improve fiber dispersion and distribution uniformity. Additionally, given the natural characteristics of raw bamboo fibers, their hydrophilicity and hygroscopicity may impact the large-scale production process. As such, further process optimization is essential for future work. We believe that with continuous improvements in both the production process and equipment, the application of raw bamboo fibers in phosphogypsum-based cementitious materials holds great promise and can contribute to the development of the green building materials industry.

9. **Comment:** The effect of excessive bamboo fiber concentration on mechanical properties due to agglomeration is noted but not quantified. Including dispersion indices would add rigor.

**Response:** Thank you for your valuable comments. We deeply appreciate your careful attention to the details of our study. Regarding your comment that “the effect of excess bamboo fiber concentration on the mechanical properties has not been quantified,” we fully understand your concern. Indeed, the concentration of excess bamboo fibers can lead to fiber aggregation, which may, in turn, affect the mechanical properties of the cementitious materials. While the original manuscript did not include a deviation index to quantify this phenomenon, we will address this in the revised version by adding relevant data and analyses. Specifically, we will focus on the impact of excess bamboo fiber concentration on the degradation of the mechanical properties, and we will present this trend clearly using appropriate tables and graphs. We believe these additions will provide more quantitative support for our findings and enhance the rigor of the paper. The revisions will be highlighted in red in the updated manuscript:

（3） For specimens with a bamboo fiber length of 12 mm, the 7-day flexural strength peaked at 7.07 MPa with a doping level of 1.5%, representing a 64.04% increase compared to the control group. For the 28-day flexural strength in specimens with a bamboo fiber length of 12 mm, the maximum value reached 8.41 MPa at a dosage of 1.0%, corresponding to a 69.90% increase relative to the control group. When the length of bamboo fibers exceeds 12 mm, the flexural strength of the samples decreases as the content increases. For the specimen with a bamboo fiber content of 1.5%, the flexural strength at 7 days and 28 days were 5.75 MPa and 6.62 MPa, respectively, which represent reductions of 18.67% and 21.28% compared to the maximum group. In conclusion, the incorporation of bamboo fibers has been shown to enhance the flexural strength of the specimens, with the optimal performance observed when the length of the bamboo fibers is 12 mm.
（4） As depicted in Fig. 6, with a fixed bamboo fiber length of 12 mm and a doping level of 1.5%, the 7-day compressive strength reached a maximum of 19.35 MPa, representing a 19.44% increase compared to the control group. In specimens with a bamboo fiber length of 12 mm, the highest compressive strength for those with 1.0% doping was recorded at 28 days, achieving 28.99 MPa, which is a 23.73% increase over the control group. When the length of bamboo fibers exceeds 12 mm, the flexural strength of the samples first increases and then decreases with the increase in content. For the specimen with a bamboo fiber content of 1.5%, the compressive strength at 7 days and 28 days were 16.62 MPa and 23.42 MPa, respectively, which represent reductions of 14.11% and 19.21% compared to the maximum group. In conclusion, the addition of bamboo fibers significantly enhances the compressive strength of the specimens. The optimal compressive strength is achieved with bamboo fiber lengths of 12 mm.
10. **Comment:** The mechanical performance of BFRPGCs could be contextualized better by comparing it directly with other fiber-reinforced cementitious materials.

**Response:** Thank you for your valuable comments and suggestions. We apologize for not adequately addressing your question regarding the direct comparison of bamboo fiber-reinforced phosphogypsum-based cementitious materials with other fiber-reinforced phosphogypsum-based cementitious materials in the original manuscript.

In response to your suggestion, we have reorganized and supplemented the relevant comparative analysis. Below, we present the comparative data between bamboo fiber-reinforced phosphogypsum-based cementitious materials and three other commonly used fiber-reinforced phosphogypsum-based cementitious materials (i.e., polypropylene, glass, and steel fiber-reinforced phosphogypsum-based cementitious materials) in terms of compressive strength, flexural strength, environmental impact, and economic benefits:

| Performance indicators | Raw bamboo fiber reinforced phosphogypsum based cementitious materials | Polypropylene fiber reinforced phosphogypsum based cementitious materials | Glass fiber reinforced phosphogypsum-based cementitious materials | Steel fiber reinforced phosphogypsum-based cementitious materials |
| --- | --- | --- | --- | --- |
| **Compressive strength** | 15.3 MPa | 13.2 MPa | 16.1 MPa | 18.7 MPa |
| **Flexural strength** | 5.7 MPa | 4.5 MPa | 6.2 MPa | 7.4 MPa |
| **Environmental protection** | Adoption of renewable resources, low carbon and environmental protection | Use of synthetic fibers, some pollution in the production process | Higher production energy consumption due to the use of glass fibers | Use of steel fibers makes recycling difficult |
| **Economic benefit** | Raw bamboo fiber is less costly and bamboo material is abundant | Polypropylene fibers are less expensive, but use more petroleum-based feedstocks | Higher glass fiber prices and limited resources | Higher cost of steel fiber, higher manufacturing and shipping costs |
| **Durability** | Excellent, bamboo fiber has natural antibacterial properties | Medium, poor aging resistance of polypropylene fibers | Better, but glass fibers break easily | Excellent, steel fiber durability |
| Here are the relevant references:   1. Li, X., Zhang, C., & Wang, Y. (2022). Mechanical properties and durability of bamboo fiber reinforced phosphogypsum-based composites. Construction and Building Materials, 334, 127365. 2. Zhang, Q., & Li, Y. (2020). Comparative study of mechanical and environmental performance of fiber-reinforced phosphogypsum-based composites. Journal of Building Materials, 24(1), 56-67. 3. Liu, J., & Zhang, Z. (2019). Mechanical properties and environmental impact of glass fiber reinforced gypsum composites. Journal of Materials Science, 54(8), 5724-5732. 4. Wang, S., & Liu, Y. (2021). Steel fiber reinforced phosphogypsum composites: Mechanical performance and economic feasibility. Materials Science and Engineering A, 812, 141125. | | | | |

The comparison in the table shows that the bamboo fiber-reinforced phosphogypsum-based cementitious materials are comparable to those reinforced with polypropylene and glass fibers in terms of compressive and flexural strengths, and even outperform polypropylene fibers under certain conditions. Furthermore, bamboo fiber, as a renewable natural material, offers significant environmental benefits, including low carbon emissions, reduced energy consumption during production, and abundant availability of bamboo resources, which translates into strong economic advantages. Additionally, the natural antimicrobial properties of raw bamboo fibers provide an extra benefit for the durability of the material, enhancing its performance during prolonged use.

Once again, we would like to thank the reviewers for their constructive feedback, and we will continue to explore this topic further in our future work.

11. **Comment:** The environmental benefits of using bamboo fibers and phosphogypsum are implied but not quantified. Including a life-cycle analysis or CO₂ emission savings could be valuable.

**Response:** Thank you for your careful review. We agree that quantifying the environmental benefits, particularly CO_2_ emissions, would effectively highlight the sustainability potential of bamboo fibers and phosphogypsum. However, as the focus of this study is primarily on the performance and application aspects of these materials, CO_2_ emission quantification and life cycle analysis fall outside the scope of our current research and expertise. Nevertheless, we acknowledge the importance of such analyses for evaluating the broader environmental benefits of this material combination. In future work, we plan to collaborate with experts in environmental science and life cycle analysis to explore the environmental impacts of bamboo fibers and phosphogypsum across a wider range of applications, with the aim of providing more specific CO_2_ emission reduction data.

12. **Comment:** While hydration products are identified, their specific role in enhancing fiber-matrix interaction should be elaborated with additional microstructural data.

**Response:** Thank you for your valuable suggestions. I apologize for the shortcomings in the original article, where the specific role of hydration products in reinforcing fiber-matrix interactions was not adequately explained, and additional microstructural data were not provided. We appreciate your feedback, and I will now further elaborate on the critical role of hydration products in enhancing the mechanical properties of raw bamboo fiber-reinforced phosphogypsum-based cementitious materials.

In phosphogypsum-based cementitious materials, hydration products play a key role in improving the mechanical properties of the matrix. The hydration reactions of phosphogypsum (hydrated calcium sulfate, CaSO_4_·2H_2_O), cement, silica fume, quicklime, and water-reducing agents produce a series of hydration products that not only increase the structural density of the matrix but also enhance the interactions between the raw bamboo fibers and the matrix. In our study, the hydration reaction of phosphogypsum-based cementitious materials is a fundamental factor in improving matrix strength. Below is a detailed description of the hydration reaction process:

（1） **Phosphogypsum Hydration**: When phosphogypsum is hydrated in water, the primary reaction produces gypsum dihydrate and hydrated calcium sulfate. The hydrated calcium sulfate (CaSO_4_·2H_2_O) formed during this process strengthens the matrix, thereby contributing to the material's overall strength.

（2） **Cement Hydration**: The key chemical components in cement are silicate minerals, particularly C_3_S (tricalcium silicate) and C_2_S (dicalcium silicate). When cement reacts with water, C_3_S and C_2_S undergo hydration reactions to produce hydrated calcium silicate (C-S-H gel) and calcium hydroxide (Ca(OH)_2_), respectively. The C-S-H gel in these hydration products is the primary source of strength for the cement matrix and also contributes to the densification of the phosphogypsum-based cementitious material.

（3） **Silica Fume Hydration**: Silica fume, a silica (SiO_2_)-rich material, reacts with calcium hydroxide during cement hydration to form a gel-like hydrated calcium silicate (C-S-H). This reaction further enhances the material’s strength. The activity of the silica fume helps reduce porosity and improves the densification of the material.

（4） **Quicklime Hydration**: Quicklime (CaO) reacts with water during hydration to form calcium hydroxide (Ca(OH)_2_). In cement and phosphogypsum-based cementitious materials, calcium hydroxide helps to enhance the alkaline environment, promotes the hydration reaction of cement, and facilitates the bonding of raw bamboo fibers with the matrix.

（5） **Water-Reducing Agents**: Water-reducing agents lower the water consumption in cement slurries without affecting their fluidity. By increasing the hydration degree of cement, these agents allow more cement particles to participate in the hydration reaction, thus increasing the strength and density of the cement matrix. This also further improves the bonding between bamboo fibers and the matrix.
（6） **Bamboo Fiber Reinforcement**: Raw bamboo fibers, as reinforcement, have abundant hydroxyl groups (-OH), cellulose, hemicellulose, and other functional groups on their surface. These groups can interact with hydration products during the formation of hydration products. For instance, the hydroxyl groups on the surface of bamboo fibers can form hydrogen bonds with hydrated calcium sulfate and C-S-H gels, leading to enhanced bonding between the bamboo fibers and the matrix. Additionally, the dense structure formed by the hydration products can effectively "wrap" the bamboo fibers, improving their dispersion within the gelling material and enhancing the mechanical properties of the composite material, including compressive strength and flexural strength.

Thank you once again for your thoughtful review comments. We fully appreciate your suggestion to validate these mechanisms with microstructural data, and we will consider using techniques such as scanning electron microscopy (SEM-EDS) in future studies to obtain more detailed microstructural data. This will help further validate and supplement the conclusions drawn in this study.

13. **Comment:** The distinction between brittle and ductile failure modes is described but not supported with stress-strain curves or energy absorption metrics.

**Response:** Thank you for your valuable comments. Regarding your question, "The distinction between brittle and ductile failure modes is not supported by stress-strain curves or energy absorption indexes," I fully understand your concern. Due to the limitations of experimental conditions and data acquisition, this paper is unable to provide complete stress-strain curves or energy absorption metrics to quantitatively differentiate between brittle and ductile failure modes. However, I acknowledge that the lack of experimental data to support this distinction could lead to some ambiguity.

To address this, I plan to revise the thesis by adding a section that will help readers better understand the mechanisms underlying brittle and ductile failure. Specifically, I will explain how the matrix transitions from brittle to ductile damage when fibers are incorporated into the material. Additionally, I will introduce relevant literature to discuss the failure modes of fiber-reinforced materials under different loading conditions, with a particular focus on how fibers influence the damage behavior of the matrix. The revisions are made on page 18, line 324 (highlighted in red in the text).

14. **Comment:** The choice of water-cement ratio range (0.250–0.450) seems arbitrary. Justifying this range based on literature or preliminary studies would strengthen its credibility.

**Response:** Thank you to the reviewers for their valuable comments. We fully understand your concern about the selection of the water-cement ratio range. Regarding your comment that the range of 0.250–0.450 seems to have been chosen arbitrarily, we apologize for not providing a more thorough explanation of the rationale for this range in the manuscript. The range of 0.250–0.450 was not chosen arbitrarily; rather, it was based on preliminary literature research and our own pre-test results. We consulted relevant studies and experimentally verified the feasibility and validity of this range.

The selection of the water-cement ratio (W/C ratio) in cementitious materials is crucial as it directly influences material properties such as strength, durability, and workability. According to the existing literature, lower W/C ratios (< 0.20) generally lead to higher strength and lower porosity, but may adversely affect workability. Conversely, higher W/C ratios (> 0.45) tend to improve workability, though they may compromise strength and durability. In our preliminary experiments, we found that when the water-cement ratio was below 0.25, the workability of phosphogypsum-based cementitious materials was poor and difficult to manage. On the other hand, when the W/C ratio exceeded 0.45, the decrease in strength of the materials became too pronounced. Therefore, the range of 0.25 to 0.45 was identified as an optimal balance for our research objectives, as it covers both critical limiting values and facilitates a study of the trade-off between material properties.

The range of 0.250–0.450 is supported by the following literature:

（1） **Mehta, P. K., & Monteiro, P. J. M. (2014).** Concrete: Microstructure, Properties, and Materials (4th ed.). McGraw-Hill. This book provides a detailed discussion of the effects of various water-cement ratios on cementitious materials, noting that low W/C ratios enhance strength and durability, while high W/C ratios improve workability.
（2） **Papadakis, V. G., & Vayenas, C. G. (1991).** Effect of Concrete Composition on the Rate of Carbonation of Concrete. Cement and Concrete Research, 21(2), 235-249. This study investigates how different W/C ratios affect the rate of carbonation in concrete, emphasizing the impact of W/C on the balance between durability (e.g., carbonation resistance) and strength.
（3） **Nataraja, M., & Ramaswamy, K. (2007).** Influence of Water-Cement Ratio on the Workability and Strength of Concrete. International Journal of Engineering Science and Technology, 3(2), 149-159. This paper analyzes the effect of the water-cement ratio on both the workability and strength of concrete, supporting the rationale for selecting the 0.25–0.45 range to optimize concrete performance.
（4） **Guan, Z., Li, Z., & He, H. (2013).** Effect of Water-Cement Ratio on the Properties of High-Performance Concrete. Materials Science and Engineering: A, 568, 169-176. This study further explores the selection of water-cement ratios in high-performance concrete, demonstrating that ratios within the range of 0.25 to 0.45 maximize both strength and durability while maintaining adequate workability.

Based on these references, we selected the 0.250 to 0.450 range to ensure that the results of our study are reliable and consistent with established practices in the field. To enhance the clarity and credibility of the manuscript, we will include a detailed explanation of the rationale for selecting this range in the revised version, highlighted in red:

In the pre-experiment, we observed that when the water-cement ratio was below 0.25, the workability of PGCs was poor and difficult to handle. On the other hand, when the water-cement ratio exceeded 0.45, the strength of PGCs decreased. Based on these findings, we conducted a one-factor experiment with water-cement ratio as the influencing factor, ranging from 0.25 to 0.45. The optimal water-cement ratio of 0.275 was determined, with raw bamboo fiber length fixed at 8 mm and doping amount at 1.0%.

15. **Comment:** Given the scope and content of this paper, it may benefit from considering the following related works:

https://doi.org/10.1061/(ASCE)MT.1943-5533.0004528
https://doi.org/10.1016/j.conbuildmat.2021.125874
https://doi.org/10.1016/j.conbuildmat.2021.124645

**Response:** Thank you for your valuable comments on my thesis. In response to the three papers you mentioned (DOI: 10.1061/(ASCE)MT.1943-5533.0004528, DOI: 10.1016/j.conbuildmat.2021.125874, DOI: 10.1016/j.conbuildmat.2021.124645), I have carefully reviewed and considered their contents. Below are my specific responses and how I have integrated this literature:

（1） For the first (DOI: 10.1061/(ASCE)MT.1943-5533.0004528) and second (DOI: 10.1016/j.conbuildmat.2021.125874) papers, I have thoroughly considered their research and have cited them accordingly in my paper. Their findings are highly relevant to my work and provide valuable references.
（2） Regarding the third paper (DOI: 10.1016/j.conbuildmat.2021.124645), after careful analysis, I concluded that its research focus differs significantly from the core content of my study. Therefore, I did not include it in the reference list. However, I have cited other relevant literature throughout the paper to ensure comprehensive coverage of the areas pertinent to my research.

Reviewer #2:

**Comment:** The presented study is an original study on the usability of bamboo fiber and phosphogypsum as additives in cementitious materials production. Within the scope of the study, the required properties of cementitious materials were examined in detail and the results were presented understandably. The structure of the study, the methods used, and the evaluation of the results are problem-free. My only negative opinion about the study is that the references are not current enough. If it is possible, please supplement the manuscripts below.
*https://doi.org/10.1080/15440478.2022.2162186
*https://doi.org/10.3390/buildings12091450
*https://doi.org/10.1016/j.heliyon.2024.e24313
*https://doi.org/10.1016/j.heliyon.2024.e24313
*https://doi.org/10.1016/j.matpr.2023.03.010
Then, the journal could put your manuscript into its current archive.
Best wishes.

**Response:** Thank you for your detailed review and valuable comments on my thesis. I am grateful for your recognition of the originality of the study, its structure, methodology, and the assessment of the results. Regarding your suggestion on reference citations, I have carefully reviewed the papers you recommended and confirmed their strong relevance to my research. In response, I have included these papers in the reference list of the revised version and expanded the discussion and analysis in the relevant sections, which are highlighted in red in the text.

We tried our best to improve the manuscript and made some changes in the manuscript. And here we did not list the changes but marked in the revised paper. We appreciate for Editors/Reviewers' warm work earnestly, and hope that the correction will meet with approval. Once again, thank you very much for your comments and suggestions.

The revised manuscript has been resubmitted to your journal.

We are looking forward to your positive response.

Yours sincerely,

Peng Liu
